# Anomaly Detection for Individual Sequences with Applications in Identifying Malicious Tools †

**DOI:** 10.3390/e22060649

**Published:** 2020-06-12

**Authors:** Shachar Siboni, Asaf Cohen

**Affiliations:** 1 Department of Software and Information Systems Engineering, Ben-Gurion University of the Negev, Beer-Sheva 8410501, Israel; 2 School of Electrical and Computer Engineering, Ben-Gurion University of the Negev, Beer-Sheva 8410501, Israel

**Keywords:** anomaly detection, individual sequences, one-dimensional time series, universal compression, probability assignment, statistical model, learning, computer security, botnets, command and control channels, NYC taxi data

## Abstract

Anomaly detection refers to the problem of identifying abnormal behaviour within a set of measurements. In many cases, one has some statistical model for normal data, and wishes to identify whether new data fit the model or not. However, in others, while there are normal data to learn from, there is no statistical model for this data, and there is no structured parameter set to estimate. Thus, one is forced to assume an individual sequences setup, where there is no given model or any guarantee that such a model exists. In this work, we propose a universal anomaly detection algorithm for one-dimensional time series that is able to learn the normal behaviour of systems and alert for abnormalities, without assuming anything on the normal data, or anything on the anomalies. The suggested method utilizes new information measures that were derived from the Lempel–Ziv (LZ) compression algorithm in order to optimally and efficiently learn the normal behaviour (during learning), and then estimate the likelihood of new data (during operation) and classify it accordingly. We apply the algorithm to key problems in computer security, as well as a benchmark anomaly detection data set, all using simple, single-feature time-indexed data. The first is detecting Botnets Command and Control (C&C) channels without deep inspection. We then apply it to the problems of malicious tools detection via system calls monitoring and data leakage identification.We conclude with the New York City (NYC) taxi data. Finally, while using information theoretic tools, we show that an attacker’s attempt to maliciously fool the detection system by trying to generate normal data is bound to fail, either due to a high probability of error or because of the need for huge amounts of resources.

## 1. Introduction

Modern computer threats are far more complicated than those that have been seen in the past. They are constantly evolving, altering their appearance and perpetually changing disguise. Indeed, while older computer viruses could be easily identified by locating known pieces of their code on the computer hard-drive or in email attachments, modern attacks are distributed, they utilize legitimate protocols and communication channels [1,2,3,4,5,6,7], and constantly change their code, servers, and attack strategies [8].

Under such circumstances, detecting unknown threats, fortiori zero-day attacks, requires new tools, which are able to capture the essence of their different behaviour (as compared to benign data), rather than some fixed signatures. Consequently, the current literature includes numerous anomaly detection techniques, which focus on detecting abnormal behaviour, rather than locating known signatures. Section 1.1 includes an overview of several such works.

Nevertheless, the currently known anomaly detection techniques are still based on a few hindering assumptions, which restricts our ability to cope with current and future attacks. First, for successful anomaly detection, one must have a good model for the normal data. That is, when learning, for example, normal HTTP traffic, one has to correctly and robustly capture the essence of such traffic, in order to identify deviations on the one hand, yet not to over-fit on the other, as this would result in high false alarm rates. Most of the classical techniques assume some statistical model for the data and, in essence, estimate the parameters of such a model. A few examples include a Markov model [4] or blending the probabilities of several Markov models using pre-defined weights [9], Auto Regressive Moving Average (ARMA) models [10], and even the use of relative entropy to decide between a few known probabilistic models [11]. In Section 1.1, we include a more detailed review of this line of works.

However, the key questions that arise are the following. What if, as detection systems designers, we do not have the statistical model for the normal data? Moreover, what if there is no such statistical model? Are memoryless features, which disregard the context of an event in the system, sufficient? What if an anomalous behaviour can only be identified by taking into account the sequence of events preceding a given point in time, and not just by a single anomalous value of a certain feature? To the best of our knowledge, works that do not assume a statistical model use compression algorithms as a black-box, aiming at identifying anomalies by differences in the compression rates or description lengths, e.g., [12,13,14,15] and several more reported in Section 1.1. Herein, we offer a different approach, calculating the probabilities of short sequences against a learned universal model, which results in high accuracy and a very short delay.

A second important aspect is the detection complexity and captures necessary for the anomaly detection system to perform properly. Modern Internet Service Providers (ISPs), for example, are required for monitoring huge amounts of data every second. Clearly, a Deep Packet Inspection (DPI) is not feasible in most circumstances. What are, then, the main features of the traffic that can be efficiently captured, yet allow for good enough anomaly detection? Using the model suggested, we show that, for example, very short, quantized sequences of timing data are enough for efficient detection.

### 1.1. Related Work

In this section, we review the most relevant previous works, and stress the uniqueness in the scheme that we suggest. The categories below are by the main methods applied.

#### 1.1.1. Information–Measures–Based Anomaly Detection

The use of information measures in general, and entropy in particular, are common in the anomaly detection literature. The celebrated work [16] used information measures, such as conditional entropy, for anomaly detection. In [17], the authors introduced Anode, which is an entropy-based network anomaly detector. The paper considered parameterized versions of entropy (e.g., Rènyi), and anomalies were identified by comparing the parameterized entropy during a given window to maximum and minimum values calculated during training. Generalized entropy measures were also considered in [18], this time using a one-class Support Vector Machine for classification. However, both works [17,18] only considered single-letter entropy measures, thus focusing on the diversity within a window, rather than a longer-term context and multi-letter measures. In fact, [19] includes a detailed survey of Entropy-based Network Intrusion Detection Systems (E-NIDS) until 2015. Recently, a single-letter entropy measure was also used in [20] to detect the failures in car components. For example, the Tsallis entropy was shown to easily identify a broken thermostat. Ref. [21] considered energy-based fault detection in the petrochemical industries. However, the work is focused on showing that a list of common process faults can indeed be identified while using fixed thresholds. A very recent review on entropy-based methods to detect anomalies in industrial environments, and rotating machinery, specifically, is given in [22]. These methods also proved useful in a linguistic context in [23], where a time series of entropies normalized by log() the size of the alphabet; hence, comparing them to a uniform distribution was used to successfully detect changes in newspapers’ content. Additionally, in the context of detecting changes in time, Ref. [24] used divergence measures, such as Kullback–Leibler (KL), between distributions of the data computed at different times in order to detect anomalies in big data streams. In [25], the authors suggested an entropy-based method to detect salient points in a crowd. This time, the entropy is used to measure the uncertainty of a particle motion direction, hence describing the chaotic degree of crowd motion. Anomalies in a matrix of such single-letter entropies are detected as salient points.

As an alternative to the Shannon entropy, Ref. [26] suggested an exponential message importance measure. This measure, in a sense, averages an exponential function of the probabilities, rather than a logarithmic one, and it is useful in data recommendation systems. However, in this paper, since detection is based on, eventually, comparing *n*-tuple probabilities to a threshold, a monotone function of the probabilities will not significantly affect the detection performance. In the same context of generalized entropy measures [17,18], Ref. [27] considered multiple orders of the Rènyi entropy for intrusion detection. In this case; however, an entropy formulation is also assumed for the intrusions. Additionally, in the context of network traffic, now for proactive defence routers, Ref. [28] used Euclidean distance between a new event log and offline-learned syslogs (represented as clusters) in order to identify anomalies. However, again, the offline procedure in the learning phase, used to transform raw data to clusters, was done on both the benign as well as the corrupted user actions.

The above works use single-letter expressions, computing either entropies or divergence measures, yet, for fixed-length distributions, single-letter or windowed, but without a continuous, long-term accounting for memory, like the one possible using LZ-parsing used herein. We improve on the above schemes in a few other dimensions as well. First, we do not assume any probabilistic model, and even do not require the convergence of empirical frequencies and empirical entropies. The suggested technique performs as good as the best finite state machine, regardless of whether the measures converge or not. If they do converge, our assigned probabilities are guaranteed to converge to the correct value. Second, the probability assignment method is highly efficient, with linear time to build. For comparison, implementing the conditional entropy in [16] is exponential in the context length.

#### 1.1.2. Parameter Estimation

As mentioned earlier, anomaly detection is intimately related to parameter estimation in probabilistic models, as, once a probabilistic model for the data is given, anomalous data can be detected if they deviate from the estimated model. Following [4,9,10,11], in [29], the authors considered representing a stochastic process while using the *d*-truncated Karhunen-Loève expansion. Clearly, this transformation can capture the memory in the data, as compared to single-letter measures. Because the expansion depends on the covariance matrix of the data, this matrix is first estimated, and then anomalies can be found if the *d*-dimensional vector, assumed as being normal, lies outside a given ellipsoid. An alternative non-parametric model estimates a local entropy measure of this representation. Again, since the local entropy depends on a distribution, it is first estimated.

Specifically in the context of computer security and intrusion detection, several recent works used the data set given in [30] (UNSW-NB15) together with novel techniques for parameter estimation. In [31], it was used successfully for intrusion detection. The decision engine in [31] assumes a Dirichlet mixture model for the multi-dimensional data (above 40 features per entry in the data set), and it is based on estimating its parameters at the learning phase. The suggested mixture model efficiently estimates the complex relations between the features. Later, a Beta-mixture model was successfully used in [32], and an enhanced system, with a novel Geometric Area Analysis, in [33]. Better and better results were achieved when more components were added in the Principle Component Analysis stage of the system, with very good results for 10 components. However, this should be compared to the algorithm herein, which operates on a time-series of a single feature.

While not in the context of parameter estimation, yet using supervised learning and decision trees on the features of the UNSW-NB15 data set, Ref. [34] proposed a two-level scheme for identification, first categorising traffic as normal or anomalous, and then deciding on the sub-categories.

In this paper, we completely skip the plug-in approach, where probabilistic measures are assumed, and their parameters are first estimated, then used, and take a different track, where such measures are not assumed to exist at all.

#### 1.1.3. Non-Parametric Models

Few works do not assume a statistical model. Generally speaking, these works use the compression rate as a single, one-dimensional feature of the data, and to achieve independence of a statistical model simply use the rates given by a universal compressor, e.g., [12,14]. A more recent example is [35], or [36], which includes a recent survey on using Kolmogorov complexity, a model-independent method, in cybersecurity, and anomaly detection specifically. The difference from our approach is critical: while [35] (or methods in [36]) uses LZ (or Kolmogorov complexity) as a black-box, and compares compression rates, thus representing normal data by a single figure, we construct a multi-letter model, which is able to assign probabilities for sequences. The compression rates can be viewed as a special case.

In [13], the authors compared compression rates, given a model vs. without it. Ref. [15] defined atypicality in the sense of being described with fewer bits in itself rather than using the (optimum) code for typical sequences. For the i.i.d. case, this requires computing the divergence between the true distribution, assumed known, and an empirical one. For a more general case, anomalies are assumed to be generated by another Finite State Machine (FSM), distinct from the one generating the normal data. Without any assumption, Ref. [15] focused on the description length and compressibility. This requires computing lengths for every subsequence of the suspected sequence in order to achieve a figure of merit ([37] extends the journal version [38] to real-valued data). In the scheme suggested herein, probabilities are directly assigned to short sequences, with one traverse through a tree. Short sequences, only about 6–10 symbols, are enough to reach a conclusion, hence causing a minimal delay, as shown in the experimental results (Section 3 and Section 6). Even better results can be achieved with a majority vote on a few sequences. The conference version of this scheme appeared in [39]. The current paper includes algorithms and results for three new applications (Section 4, Section 5 and Section 6), together with a detailed threshold analysis, and analysis on the complexity required to attack this scheme (Section 7).

#### 1.1.4. Deep Learning for Anomaly Detection

In recent years, there has been a surge in the use of deep learning approaches for anomaly detection, and specifically for traffic analysis. Indeed, with enough labeled instances, deep neural networks can successfully handle huge amounts of multi-dimensional data. A few celebrated examples are [40], which also used the UNSW-NB15 data set, and proposed an anomaly detection technique for industrial control systems that is based on deep learning. The method in [40] is based on a feed-forward neural network, with stochastic gradient descent back-propagation for training, yet with the addition of a deep auto-encoder to reduce dimensionality. The results are excellent when compared to the mixture approach presented in [31], and support vector machines or other deep learning approaches. However, this method uses all features of the data initially, and requires the complexity of neural networks and back-propagation. Hence, it is a complex, multi-dimensional detection system, with excellent results on this data set. New learning methods also used recurrent neural networks in [41], or the excellent results, yet with 10 hidden layers and using the full data set for training, as given in [42]. Note that these methods require high computational complexity in the learning phase. For example, the work in [41] was implemented using TensorFlow, a large-scale and diverse Machine Learning environment, or [43,44], which used Apache Spark on a cloud. Still, exceptional performance can be achieved with deep neural networks. Very recently, Ref. [45] also used an auto encoder to balance the training data, reduce dimensionality, and initialise the weights of the hidden layers. In the same context, dividing the training set into several subsets to improve the balance in the training set was found to be useful in [46]. An imbalance between benign and anomalous data was also successfully treated in [47]. Recently, comprehensive surveys on deep learning for intrusion detection are given in [48,49]. However, note that, in the context of this work, there are two critical differences between the algorithm suggested herein, and the deep learning approaches above. First, we offer a simple model to build and test, operating on one-dimensional, single-feature sequences, usually with a defined notion of time (see, e.g., Section 6). This should be compared to the systems above, operating on multi-dimensional data (the data set in [30] has above 40 features), with some pre-processing for feature selection [50,51] and a single-entry per flow. Second, deep learning usually demands substantial computations, with iterative learning (e.g., back propagation) and sometimes dedicated cloud platform or even hardware, as mentioned above. This should be compared to a one-pass, linear time algorithm to build the model that is suggested herein.

Finally, note that, when the ordering of the data is the key feature to discriminate between normal and anomalous events, permutation entropy was found to be useful. This is the case, for example, in climate data [52]. However, in many computer security applications, abnormal data may include the same order of events, with only slight variations in timing. We elaborate on computer security applications in Section 3.1 and later in the paper. Anomaly detection is also intimately related to change-point detection [53], especially when the parameters after the change are unknown, though, most of the literature focuses on i.i.d. sources, assumes a time when the change occurred, and calculates the trade-offs between false alarm and detection delay.

### 1.2. Main Contributions

We suggest a universal anomaly detection technique for one-dimensional individual sequences, which does not require any a-priori information on neither the normal behaviour patterns nor the abnormal ones, yet efficiently learns the normal behaviour and generates a statistical model to which new instances can be compared. The technique is based on the LZ algorithm, which is an optimal universal compression algorithm. In fact, when applied to stationary and ergodic sources over finite alphabets, its compression ratio converges to the entropy rate and, from the model, it implicitly generates one asymptotically optimal probability assignment. Using the induced assignment, we rigorously define the statistical model, which represents the normal behaviour and offers a mechanism to test new, unknown sequences, while using this model. Moreover, the suggested technique inherits key useful aspects of the universal compression algorithm, that is, it performs optimally (in terms of estimating the model), even on individual sequences, i.e., when there is no statistical model, and is extremely efficient to implement in practice, with linear time to build and test.

Furthermore, the new anomaly detection system offers a new look on the way to use data in the classification process, offering the context of the data sequence as the key characteristic used in the classification, rather than actual values. That is, the suggested system does not rely on memoryless features of the data, such as frequencies of occurrences of certain events. In contrast, it builds a context tree for the learned data, while taking memory and long-term, joint distribution of the events into account.

We evaluate the anomaly detection technique with real-world test cases and give the required processing and detection algorithms for each. The first is identifying Botnets’ C&C. In this case, we use timing data as the single feature. This allows for us to be both protocol-independent and encryption-independent. Moreover, it allows for us to suggest a system that is immune to various hiding techniques. Again, the key concept, however, is not to use the actual values in the timing data, but, rather, the context of the different timings. The results on real network traces clearly show that the suggested model is a favorable solution, with excellent results in terms of low false alarm and high detection rates, yet with only moderate (linear time) complexity and no deep packet inspection. To make an even stronger case, we give two additional computer security test cases, malicious tools identification via system calls and data leakage identification, as well as the results of the algorithm on the benchmark NYC taxi data, where the algorithm successfully identified all known anomalies, with very few false alarms. All use a single-feature, one-dimensional data set to identify the anomalies. Moreover, we include a detailed threshold analysis, together with implementation of methods that allow for setting the detection threshold without any labeled data, a highly desired objective for anomaly detection algorithms in real-world applications.

Finally, we consider possible prevention strategies that attackers can use against the suggested system, and prove its robustness by arguing that such strategies would either fail or require huge amounts of data and massive learning, which are usually unrealistic for regular Bots.

The rest of the paper is organized, as follows. Section 2 first gives a short background on classification and anomaly detection, and then describes, in detail, the suggested algorithm: from universal probability assignment for individual sequences, through the learning phase, to testing for anomalies and the performance metrics used. Subsequently, Section 3, Section 4 and Section 5 describe three network security test cases: Botnets, Malicious system tools and Data Leakage, respectively. For all three, we describe the pre-processing that is needed to apply the suggested algorithm, apply it, and present results on real network traces and system calls. Section 6 does the same for the benchmark NYC taxi data set. Section 7 discusses prevention strategies that an attacker can use and proves the system’s robustness. Section 8 concludes this paper.

## 2. Universal Anomaly Detection for Individual Sequences

We first give a brief overview of classification and anomaly detection. We then describe how to construct a universal probability assignment for individual sequences, and use it as a building block for anomaly detection. This description is followed by formally presenting the learning and testing phases of a general system. We conclude the section with a short discussion on performance metrics and practical constraints.

Throughout, calligraphic letters denote a finite alphabet, e.g., S, lower case letters denote symbols, *s*, while boldface denotes sequences, s. It will be beneficial to denote phrases, short sequences of symbols, with a capital letter, e.g., *S*.

### 2.1. Classification and Anomaly Detection

Classification refers to the problem of labeling unknown (new) instances to the most appropriate class among a set of (known) predefined classes. When the underlying probability distributions for the classes ${\left\{P_{i}\left(\cdot \right)\right\}}_{i=1}^{M}$ are known, and we wish to decide which generated a given data sequence y, a decision rule of the form

$$\hat{i}={\mathrm{argmax}}_{1\leq i\leq M}P_{i}\left(y\right)$$

is optimal in the sense of minimizing the probability of error [54].

However, in unary-class classification, information is only available on one type of instances, namely, there is only one class, P(·). Nevertheless, the goal is either to identify instances belonging to this class, or, taking the opposite viewpoint, referred to as anomaly detection, in order to identify instances that do not belong to the class. Specifically, assume, for now, a given probability distribution (of a single class) P(·). We refer to this class as normal. In anomaly detection, the goal is then to identify whether a new data instance y belongs to the normal class, or, alternatively, is anomalous. Because, in most applications, the anomalous instances are threats that one wishes to identify, we refer to a correct identification of an anomalous y as detection and for an incorrect identification of normal data as false alarm. The optimal decision rule in terms of maximizing the detection probability given a fixed false alarm probability (in the Neyman–Pearson sense) is to compare P(y) to a threshold and decide that y is normal if P(y) is above the threshold and anomalous otherwise [55]. The threshold is determined according to the required false alarm probability.

In practice, the probability distributions governing the data (either multiple classes or a single one) are unknown, and there is only a limited amount of data to learn from. Furthermore, in most security-related applications, only few, if any at all, anomalous instances to learn from exist, yet more instances of normal behaviour are available. This asymmetry strengthens the need to take the anomaly detection approach in such circumstances, which is, build a behavioural model that is only based on normal instances, and classify any instance deviating from that model as anomalous [56]. Thus, a reasonable approach is to estimate the probability distribution of normal data while using the previously observed sequences and the resulting estimate, $\hat{P}\left(\cdot \right)$, in the detection algorithm.

### 2.2. Universal Probability Assignment for Individual Sequences

The above estimation problem is structured and relatively easy to solve if a statistical model for the normal data is given. E.g., if the normal data is known to be generated by an i.i.d. or a Markovian model of a certain order, then, given the data sequences (instances of the above distributions) only a few parameters should be estimated to construct $\hat{P}\left(\cdot \right)$. However, this is not the case in this paper. Herein, we wish to eliminate all assumptions on the normal data, even the assumption that the sequences are governed by some stationary and ergodic source. We assume the data constitutes of individual sequences, that is, deterministic sequences with no pre-defined statistical model at all. We suggest an anomaly detection technique for the most general case, where no underlying statistical model is given.

To do this, we build on the relation between prediction of discrete sequences and lossless compression [57], and harness the information measures used for universal compression algorithms in the anomaly detection procedure. The Lempel Ziv algorithm [58], LZ78, is a universal compression algorithm with a vanishing redundancy. Consequently, it can also be used as an optimal universal prediction algorithm [57], while using the appropriate probability assignment. In the context of classification, it was also used in [59] for typist identification based on keyboard events and in [60] for English text, music pieces, and proteins classification.

#### 2.2.1. The LZ78 Compression Algorithm

For a given sequence of data symbols, a dictionary of phrases parsed from that sequence is constructed based on the incremental parsing process, as follows. At the beginning the dictionary is empty. Subsequently, during each step of the algorithm, the smallest prefix of consecutive data symbols not yet seen, i.e., which does not exist in the dictionary, is parsed and added to the dictionary. By that, each phrase is a unique phrase in the dictionary, which might extend a previously seen phrase by one symbol.

Given a sequence $s=(s_{1}s_{2}\ldots s_{n})$ over a finite alphabet S, a parsed phrase, *S*, is the smallest prefix of consecutive data symbols that has not been seen yet. This can also be considered as suffix concatenation of symbol $s_{i}$ (from the sequence) with a previously seen phrase $S^{\prime }$ (from the dictionary), i.e., $S=(S^{\prime }s_{i})$. A dictionary, *D*, is a collection of all distinct phrases parsed from a given data sequence s, i.e., $D=\{S_{1},S_{2},\ldots ,S_{i}\},i\leq n$. For example, the sequence aabdbbacbbda is parsed as a|ab|d|b|ba|c|bb|da|.

A common representation of the dictionary is a rooted-tree, where each phrase in the dictionary is represented as a path from the root to an internal node in the tree according to the set of symbols the phrase consists of. Leaf-nodes are added as suffix for each phrase in the tree.

#### 2.2.2. Statistical Model for Individual Sequences

**Figure 1:**
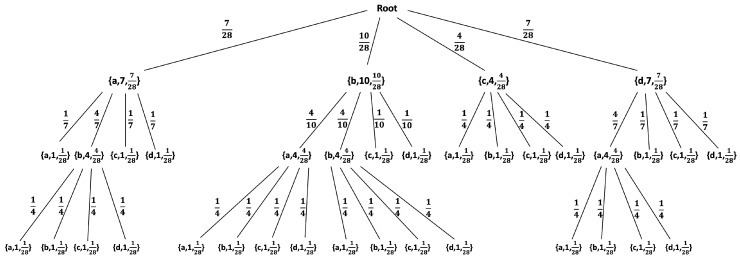
A statistical Model for sequence “aabdbbacbbda”. Each node in the tree is represented by the 3-tuple {symbol, counter, probability}. The probabilities of edges connected directly to the root are equal to the appropriate root-children’s counter divided by the total number of leaf-nodes, *i*, at each step of the algorithm.

For any individual sequence, without any assumption on its structure or underlying statistical model (or lack thereof), a statistical model can be defined during the construction of a phrase-tree [57]. At the beginning, an initial tree is constructed, including only a root node and *k* leaf-nodes as its children, where *k* is the size of the alphabet. Subsequently, for each new phrase parsed from a sequence, the tree is traversed, starting from the root, following the set of symbols the phrase consists of, and ending at the appropriate leaf-node. Once a leaf-node is reached, the tree is extended at this point by adding all the symbols from the alphabet as immediate children nodes to that leaf, which makes it an internal node. Each node in the tree, except for the root node, maintains a counter, where each leaf-node’s counter is set to 1 and each internal node’s counter is equal to the sum of its immediate children’s counters, in order to define a statistical model. For a probability assignment, as all leaf-nodes’ counters are set to 1, they are thus assumed to be uniformly distributed with a probability 1/i, where *i* is the total number of leaf-nodes. Each internal node’s probability is defined as the sum of its immediate children’s probabilities, which also equals the ratio between its counter and current *i*. The probability of an edge is defined by dividing the nodes’ probabilities. The probability of a phrase $S_{i}\in D$ is calculated by multiplying the probabilities of the edges along the path defined by the symbols of $S_{i}$. For example, Figure 1 demonstrates the resulting statistical model for the sequence “aabdbbacbbda”.

When computing the probability of a phrase given a fixed statistical model, note that, for each phrase, there is a specific node in the tree whose probability represents the probability of that phrase. For instance, based on Figure 1, $P\left(ba\right)=\frac{10}{28}\times \frac{4}{10}=\frac{4}{28}$. However, if, during the traversal of a longer sequence, a leaf-node is reached before all of the symbols of a sequence are finished, the traversal returns to the root and continues until all of the symbols of that sequence are consumed [59]. For example, the probability of the sequence “bdca” given the statistical model above is defined as the probabilities multiplication based on the path: Root→b→d→Root→c→a, and is calculated as $P\left(bdca|M_{aabdbbacbbda}\right)=\frac{10}{28}\times \frac{1}{10}\times \frac{4}{28}\times \frac{1}{4}=\frac{1}{784}$. Thus, given a sequence s, from which a model $M_{s}$ was built, and a sequence y over the same alphabet, we define by $P(y|M_{s})$ the probability assigned to y by $M_{s}$. In the context of Section 2.1, given the real data s, the model $M_{s}$ will constitute the result of the learning process, and $P(\cdot |M_{s})$ will serve as the estimate $\hat{P}\left(\cdot \right)$ in the detection process.

### 2.3. An Anomaly Detection System Via Universal Probability Assignment

#### 2.3.1. Preprocessing and Quantization

To use the estimate $P(\cdot |M_{s})$ in practice, the data from which the model is created should be represented as a one-dimensional, discrete sequence over a finite alphabet. Hence, we focus on a single-feature data sequence. Excellent detection capabilities can be achieved even on a single feature, as shown in the sequel. This is since the suggested algorithm captures the context of the events. In other words, what matters the most is not necessarily a specific value of a feature, but, rather, the sequence of values and their relations. For example, when a human browses the web, her or his browsing creates a sequence of events, with distinct timings, and, more importantly, distinct sequences, governed by the time that it takes the human being to interact with a website, and by the time that it takes a web server to respond. However, when a Bot uses an HTTPS connection as a disguise, while any single event it generates may not be anomalous, the context of several events, influenced by the fact that both the client is not human, and the server is not a legitimate web server, is, in fact, significantly different than the context in human browsing. The same applies to other features. E.g., the sequence of applications used by a user in a regular day at the office might be different when compared to the sequence used by a compromised user, whose identity has been stolen. Clearly, the suggested method can be simultaneously applied on multiple features, at the price of high dimensionality.

While the tree construction is linear in the length of the sequence, it is exponential in the alphabet size. Hence, sequences are quantized before learning or testing. For *k* quantization levels, a set of *k* centroids $\left\{c_{1},c_{2},c_{3},\ldots ,c_{k}\right\}$, is used. The centroids are extracted from the available data during the training phase. Clearly, both the number of centroids, *k*, and the method of quantization affect the overall results. On the one hand, a small alphabet size (small *k*) results in low complexity for both learning and testing, and a robust model, without over fitting. On the other hand, a small alphabet size might group together different values (e.g., time differences between network events) or types of events in the system (e.g., different system calls) under the same symbol, possibly loosing some of the the data (e.g., treating minor differences as equal, hence loosing subtle changes that might reflect distinct phenomenons), depending on the data of the specific application. However, it is important to note that the essence of the detection procedure that we suggest is not in finding a single anomalous symbol, but, rather, finding a string of symbols that represent an out-of-context behaviour. I.e., a whole string of low probability given the learned model. Hence, small changes in the parameters of the quantization do not necessarily have a huge impact on performance. Still, fine tuning should be done. This fine-tuning is easily done during training. In Section 3.3, where the sequences on which learning (and later, testing) was performed were time differences between network events, a simple uniform quantization was found to be useful. However, in Section 4, where the sequences represent system calls of a give process, the values were quantized based on functionality. The number of quantization levels (centroids) also has a small effect on performance. This is also demonstrated in Section 3.3.

#### 2.3.2. Learning and Testing

**Figure 2:**
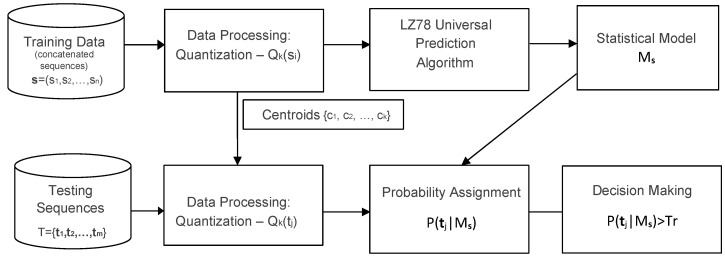
A Classification Model based on the LZ78 universal compression algorithm and its associated probability assignment.

In the learning phase, available data of normal traffic is preprocessed and an LZ78 probability assignment tree is built, to serve as the statistical model for the normal data. In the testing phase, or the actual operation of a detection system, new, unclassified data arrives. These data go through the same preprocessing, resulting in quantized sequences over the same alphabet. The sequences are then tested against the model. Figure 2 depicts the key building blocks.

Specifically, in the learning phase, an LZ78 statistical model is built according to the algorithm in Section 2.2, based on a given training set of discrete, quantized sequences over a finite alphabet S of size *k*. Training is done only on normal, benign traffic. In practice, one might not have a long enough sequence from a single, normal instance. Thus, in all cases tested for this paper, normal sequences were concatenated together to generate one long sequence, s, from which the tree was built.

In the testing phase, first, each suspected testing sequence is separately quantized using the same quantization method and the same set of centroids $\left\{c_{1},c_{2},\ldots ,c_{k}\right\}$, which were used in the learning phase. Subsequently, the probability of each suspected sequence (testing sequence) $t_{j}$, from a given testing set $T=\{t_{1},t_{2},\ldots ,t_{m}\}$ is estimated based on the constructed statistical model and classified respective to a predefined threshold $T_{r}$. Namely, the probability of each testing sequence, $\hat{P}\left(t_{j}\right)=P\left(t_{j}|M_{s}\right)$, is computed by traversing the tree. Testing sequences for which $\hat{P}\left(t_{j}\right)$ is greater than or equal to $T_{r}$ are classified as normal (as they “fit” the model), while a lower than threshold value is classified as anomalous.

It is important to note that for the testing phase the design of the classifier has a few degrees of freedom, which are based on the required trade-off between complexity, accuracy, and the availability of data. That is, while the optimal decision should be made based on a long enough sequence, in practice, one might combine a few decisions together, based on a few sequences that are known to belong to the same suspect. For example, when a server is suspected in acting as a C&C server, one might test a few sequences of data from or to the server, even if belonging to different sessions, and make a decision based on all results together.

#### 2.3.3. Performance Evaluation and Practical Constraints

The performance of the classifier is measured by the false alarm and detection probabilities (also known as False Positive, FP, and True Positive, TP, rates), and is usually demonstrated using a Receiver Operating Characteristic (ROC) curve. The false alarm probability (or ratio) reflects the number of negative (in this case, normal) instances incorrectly classified as positive (anomalous) in proportion to the total number of negatives in the test, whereas the hit detection ratio measures the proportion between the number of positive instances correctly classified and the total number of positives in the test. Each value of the threshold results in a point on the ROC curve. Changing the threshold changes the trade-off between the two probabilities. In our case, the range was $\left[\min_{j}\left(\hat{P}\left(t_{j}\right)\right),\max_{j}\left(\hat{P}\left(t_{j}\right)\right)\right]$.

Finally, the model above includes a single pass process of learning and modeling. It builds a single LZ tree that serves as the statistical model. However, the LZ tree can be updated or enhanced over time. For this, we refer the reader to a few adaptive-LZ techniques, which allow fast rebuilding of the tree, e.g., [61]. Moreover, the current literature also includes algorithms for fast and parallel construction of the tree, e.g., [62].

## 3. Botnets Identification

In this section, we introduce the key test case. We first briefly overview Botnets’ architecture and then describe how the algorithm in Section 2 was applied and its results.

### 3.1. Botnets: Architecture and Existing Detection Mechanisms

A Botnet is a logical network of compromised machines, Bots, which are remotely controlled by a Botmaster using a C&C infrastructure. The compromised machines can be any collection of vulnerable hosts, e.g., computers, mobile-phones, or tablets. The C&C channel is used by the Botmaster to send commands (e.g., launch a Distributed Denial-of-Service, DDoS, attack) or receive back information (e.g., credit card numbers). Indeed, a majority of massive cyber-attacks today are conducted by Botnets, including spamming, fraud, and identity theft, etc. Because the C&C traffic is usually assimilated to benign traffic, using anomaly detection techniques is a key method to identify it. We will use Botnets’ C&C and related problems as the main test cases for the anomaly detection algorithm in this paper.

Due to the fact that the C&C channels are the only way that the Botmaster can communicate with its Bots, they can be considered as the weakest link of a Botnet, as blocking them renders the Botnets useless. Thus, Botnets use common communication protocols as their C&C, including IRC [1,2], HTTP [2], Peer-to-Peer (P2P) [3], and DNS [6] in order to mask their activities and bypass defense mechanisms such as firewall. Recently, Botnets also adopted social networks as the underlying C&C [7]. However, while it is tempting to develop protocol-specific methods to detect Botnets, attackers constantly improve their C&C infrastructures and develop new evasion capabilities, including changing signatures of the C&C traffic, employing encryption and obfuscation in order to deceive detection systems.

Techniques to detect Botnets are either signatures-based ([63]) or use anomaly detection models [8,64,65]. Signature-based techniques use a known signature of the Botnet, hence they are are prone to zero-day attacks and require a constant update of the signatures. Anomaly-based detection techniques, on the other hand, aim to detect anomalies in network traffic or system behaviour, which may indicate the presence of malicious activities. A basic assumption when using anomaly detection is that attacks differ from normal behaviour. Thus, traffic analysis is used on both packet and flow levels, when considering metrics, such as rate, volume, latency, and timestamps in order to identify anomalous data. For example, BotSniffer [2] and BotMiner [66] are based on traffic analysis. The former was designed for IRC- and HTTP-based Botnets, while the latter was designed as protocol independent detection, which requires no prior knowledge. However, both systems rely on DPI techniques; hence, they are less suitable for on the fly analysis of large amounts of traffic. Ref. [67] suggested that a periodic behaviour indicates Botnet activity. Ref. [68] presented a detection system that considers high-level statistical features of C&C, which were extracted from known Botnets in a controlled environment, thus limiting the ability to detect new types of attacks.

### 3.2. Data Set and Pre-Processing

The data set used contains high-level real-world network traces provided by an Internet security company (in order to maintain the confidentiality of the company’s customers, the name of the company is withheld). The data set consists of 3,714,238 client-server transactions, taken during a time window of approximately three hours. The whole data set is available at [69]. Each client, denoted by ‘cid’, might connect with several hosts (web-servers), denoted by ‘hid’. On each transaction, data are sent both by the client and the host. This defines communication pairs Client-Host (denoted CID_HID). Each transaction is labeled as either legal, denoted by ‘good’, for normal traffic generated by the client, or illegal, denoted by ‘hostile’, that is, Bot traffic. Labeling was done by the security company’s experts based on well-known black-lists. Note that these labels are not used during the classification process. They are used only in the validation phase. Note also that flows from a single client may consist of both ‘good’ flows reflecting legitimate data traffic generated by the client itself, as well as ‘hostile’ flows that are generated by a Bot installed on the client. In contrast, a server (host) has only flows with the same label, that is, if a host was labeled as a C&C infrastructure then all of its transactions are considered to be malicious. The ‘Client’ and ‘Host’ definitions represented two different perspectives of the data set. On the one hand, one can examine the events occurring in the network from the client point of view, and on the other from the host point of view, as will be shown in Section 3.3.

Table 1 exemplifies the structure of the data set. Each transaction is represented by a single record, which consists of the following fields: ‘time’, referring to the time that the transaction took place; ‘time-taken’, is the total time that the transaction took; ‘cs-bytes’ and ‘sc-bytes’ fields represent the total bytes sent by the client/server(host) to the server(host)/client during the transaction, respectively; ‘mime-type’ denotes the Internet content type of the transaction, such as: plain text, image, html page, application, etc.; ‘cat’ is the category of the transaction—‘good’ or ‘hostile’; and, the ‘hid’ and ‘cid’ fields refer to the host-index (Internet site, web-server) and client-index respectively. Again, to protect the identity of the company’s customers, these indices were given arbitrary. However, some malicious sites are identified by their domain name, e.g., ‘hotsearchworld.com’ or ‘blitzkrieg88.bl.funpic.de’.

The processing of the data included serialization and feature extraction: the data set is split into flows based on CID_HID connections, as illustrated in Table 1 with respect to flows 486_52 and 9_49 (marked in gray). A ‘Flow’ is a sequence of transactions of the same communication pair CID_HID sorted by time and with the same label, either ‘good’ or ‘hostile’. In total, there are 19164 flows labeled as ‘good’ and only 65 ‘hostile’ flows (0.338%). This indicates an imbalance, where most of the transactions are legal and only a small fraction is illegal. However, this is characteristic of real network traffic behaviour. Next, selected features are extracted from each transaction. As explained in Section 2, the goal is to achieve a discrete time, finite alphabet sequence, on which learning and testing can be performed. We focus on low-level features of the data, such as timings or sizes. Such features are easy to collect, even from encrypted flows. Nevertheless, as will be demonstrated in Section 3.3, such features suffice for very robust identification of Botnets’ C&C.

#### Preprocessing of Flows before Learning and Testing

A data sequence is a series of events from a flow between a specific client and a specific host. The *i*th Network Event, denoted by $e_{i,xy}$, is a data transaction between client *x* and host *y* and it is defined by the tuple

$$e_{i,xy}=\left(t_{i},tt_{i},csb_{i},scb_{i},x,y\right),$$

where $t_{i}$ is the time event $e_{i,xy}$ occurred; $tt_{i}$ is the duration of event $e_{i,xy}$; $csb_{i}$; and, $scb_{i}$ are the total number of bytes that were sent by client *x* to host *y* and by host *y* to client *x*, respectively. A Network Flow, denoted by $f_{xy}$, is series of network events between client *x* and host *y* sorted by their time of occurrence, $t_{i}$. That is,

$$f_{xy}=\left\{e_{1,xy},e_{2,xy},\ldots ,e_{n,xy}\right\}.$$

For learning and testing, a single feature is used. For example, timing data can be characterized by the difference between two consecutive events of the same flow, denoted by Time-Difference (TD) and defined by

$$TD_{i,xy}=e_{i+1,xy}\left(t_{i+1}\right)-e_{i,xy}\left(t_{i}\right),$$

where $e_{i,xy}\left(t_{i}\right)$ refers to the first entry in $e_{i,xy}$, the time that the event occurred. A different perspective is the total time that the event took, denoted by Time-Taken (TT) and defined by

$$TT_{i,xy}=e_{i,xy}\left(tt_{i}\right).$$

Similarly, one can focus only on sizes, e.g., Client-Server-Bytes (CSB) and Server-Client-Bytes (SCB) and, respectively, define $CSB_{i,xy}=e_{i,xy}\left(csb_{i}\right)$ and $SCB_{i,xy}=e_{i,xy}\left(scb_{i}\right)$. The single-feature data sequence is a serialization of one of the above features, e.g., with respect to Time-Difference, a sequence is defined as:

$$f_{xy,TD}=\left\{e_{2,xy}\left(t_{2}\right)-e_{1,xy}\left(t_{1}\right),\;e_{3,xy}\left(t_{3}\right)-e_{2,xy}\left(t_{2}\right),\ldots ,e_{n,xy}\left(t_{n}\right)-e_{n-1,xy}\left(t_{n-1}\right)\right\}.$$

Clearly, the above procedure results in a sequence over a large alphabet. For example, times may be given with a very high precision. Hence, quantization is performed (several quantization algorithms were tested).

### 3.3. Experimental Results

For the experiments, the flows were randomly selected from the data sets, from both ‘Client’ and ‘Host’ perspectives, and divided equally between the training and testing phases. Hence, ROCs are only based on newly seen data). We first tested which single-feature achieves the best results. The system was then optimized while using this feature alone. The best results, in terms of optimal threshold and ROC-AUC (Area Under Curve), were achieved using the Time-Difference (TD) representation of the data sequences along with the ‘Uniform’ quantization. Thus, we focus on this feature. To better understand why TD was superior, consider a legitimate web surfer compared to a hostile connection using HTTP only as a C&C channel. While the surfer must have a reasonable behaviour in the time domain, affected by the times that are required to read a page, the times required for the server to respond, etc., a C&C channel might behave differently, without, for example, a reasonable response time from the server as it only collects data from the bots, and the “GET” messages are used solely to transmit information.

**Figure 3:**
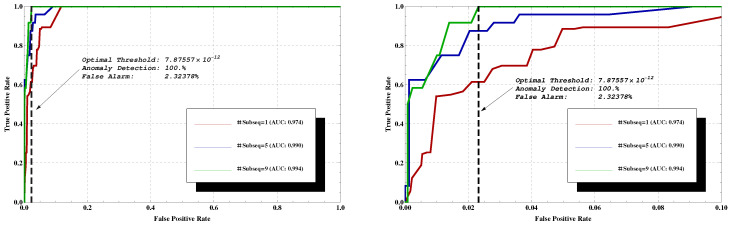
Testing ‘Majority Vote Classification’ using TD feature and ‘Uniform’ quantization method by considering ‘Clients’ type of flows. (**Left**): Receiver Operating Curve; (**Right**): Zoom in on the upper left corner. First, each testing sequence partitioned into several sets of subsequences, denoted as #Subseq in the graph, and the decision made per set of subsequences, where better results were achieved for higher subsequences definition.

Using TD, the threshold for 100% detection results in 11.75% false alarms. However, this is when only a single, short sequence is tested. A majority vote for several sequences within the flow can be used to further improve the results. Each data segment is partitioned into several subsequences of length 10. The classification is done based on the majority of these subsequences’ estimations, which results in better classification performance. For example, an AUC of 0.994 and false alarms rate of 2.32378% are achieved while using a threshold of 7.87557 × 10${}^{-12}$, as illustrated in Figure 3. This is obtained at the cost of higher detection time per data segment, of course, as using only one subsequence per data segment the decision is made immediately, while using nine subsequences causes delay.

Note that, as each point on the ROCs in Figure 3 corresponds to a different threshold value, it results in a different confusion matrix. For example, the point (0.023,1) on the green curve corresponds to the confusion matrix $\left(\begin{array}{cc} TP & FP\\ FN & TN \end{array}\right)=\left(\begin{array}{cc} 1 & 0.023\\ 0 & 0.977 \end{array}\right)$, while (0.02,0.87) on the blue curve corresponds to $\left(\begin{array}{cc} TP & FP\\ FN & TN \end{array}\right)=\left(\begin{array}{cc} 0.87 & 0.02\\ 0.13 & 0.98 \end{array}\right)$.

**Figure 4:**
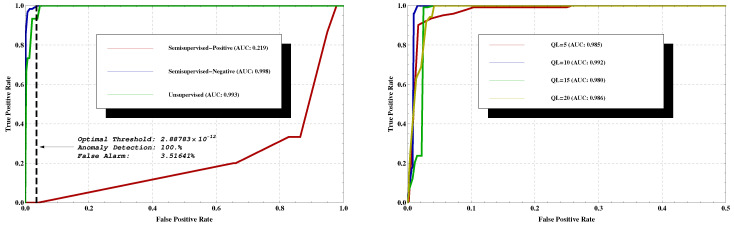
(**Left**) Testing ‘Training Modes’: Semisupervised-Negative, Semisupervised-Positive and Unsupervised modes, using TD feature and ‘Uniform’ quantization method with respect to ‘Hosts’ type of flows. The classifier achieves the best results of AUC = 0.998 with 100% detection and 3.51641% false alarms for Semisupervised-Negative training mode and the worst results of AUC = 0.219 and ∼98% false alarms for 100% detection in the case of Semisupervised-Positive training mode. (**Right**) The effect of the number of quantization levels on performance. QL refers to the number of centroids used. QL =10 achieved the best results in terms of the area under the curve, with AUC = 0.992, as depicted by the blue line in the figure.

The above results were obtained from a ‘Client’ point of view, while using semisupervised training. Next, we examine the ‘Host’ point of view, as well as differentiate between these three important aspects of the learning process: (i) ‘Semisupervised-Negative’, in which one has only normal data sequences during training, clear of anomalies. (ii) ‘Semisupervised-Positive’, which only takes into account anomalous sequences during training. (iii) The most realistic scenario, ‘Unsupervised’ where the training set consists of both normal and a few anomalous sequences as well. This is typical in most cases of interest, where one has training data, but it is unlabelled, and all that is known is that it is mostly normal. When learning using such data, the LZ tree will contain phrases which might correspond to anomalous data, but, as the majority of the data are normal, the tree will still be biased towards normal behaviour, which is the desired structure. We refer the reader to Figure 4 (left) for the results. Clearly, trying to learn only from the anomalous behaviour fails (the red curve—AUC = 0.219), as there are only few samples, and C&C traffic may differ significantly for new bots on which the system was not trained. However, the key finding is that when learning is done using noisy data, which includes some C&C traffic besides the normal one, there is no significant degradation in performance. That is, in the ‘Unsupervised’ training mode, the classifier achieves very good results, despite the fact that the underlying datasets used in the training phase contain both normal and few anomalous sequences. ‘Semisupervised-Negative’ achieves the best results of AUC = 0.998. Additionally, in Figure 4 (right) is the analysis for selecting the best *k* quantization levels (abbreviated as QL). It was found that QL = 10 achieved the best results in terms of the area under the curve (AUC = 0.992, blue line in the figure). Note that other values for k do not significantly change the results.

**Figure 5:**
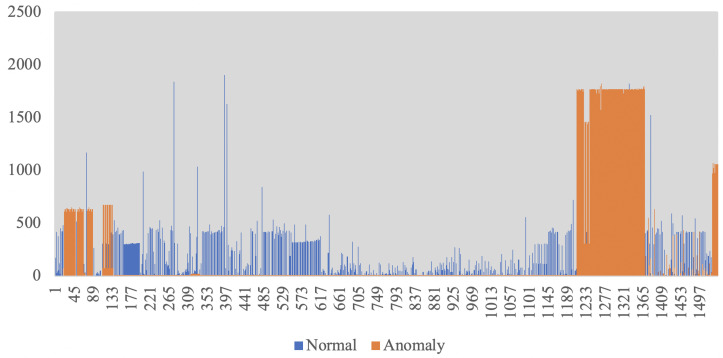
A simple example of time-differences ($TD_{i}$) for both normal and anomalous sequences. In this simple example, the normal traffic is characterized by variable values, which reflect standard network traffic. For example, a user surfing the web. The anomalous data are characterized by fixed values. This reflects the behaviour of a simple C&C channel, where the bots connect on specific times, for a specific time frame. For more complex anomalous behaviour, see also Section 6.

Finally, for a concrete example, examining flows 6_3, 6_14, and 9_1 under TD, ‘Uniform’ quantization, and a threshold of 2.88783 × 10${}^{-12}$ (obtained from the last test case), we found that all of these flows are classified as anomalies. From a client point of view, this indicates that clients 6 and 9 are infected by a Bot program, and from a host perspective this implies that hosts 1, 3, and 14 act as the command and control servers. Our findings were confirmed by examining these three servers with a list of the actual domains corresponding to the host. Server 1 is known as ‘hotsearchworld.com’, Server 3 is ‘blitzkrieg88.bl.funpic.de’, and Server 14 has IP address of 209.123.8.198, which was black-listed. Figure 5 includes a simple example of normal vs. anomalous data.

### 3.4. Threshold Analysis

In most anomaly detection applications, labeled data are scarce, if any exist; hence, it is not clear how to set the threshold for detection. Indeed, threshold detection is a critical aspect in anomaly detection solutions. For the results in this paper, two major directions were followed. One that involves labeled data, and one that does not. For each direction, we present the resulting threshold and detection performance. In this section, we first describe how the threshold was set for the above results, with labeled data. Subsequently, we discuss a method to set it without labels. Moreover, in Section 4.1, we describe another approach, which is based on Extreme Value Theory [70,71], in order to set a threshold without labeled data. We also use the same approach successfully in Section 6.1. Both cases show that, while using the suggested system, an efficient threshold can be set without labels. Clearly, labels allow for fine tuning to an even greater precision.

**Figure 6:**
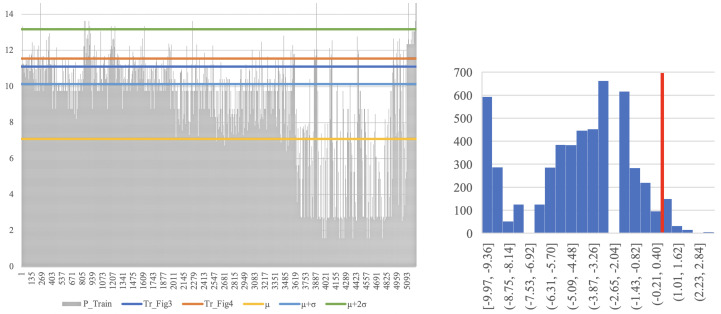
Threshold analysis. (**Left**) Probabilities of training sequences and the thresholds used from the testing phase, referred as Tr (in blue and orange). X axis refers to sequence numbers, while Y depicts the probability on a $-\log_{10}$ scale. The threshold levels decided with labeled data are between the μ+σ and μ+2σ levels (light blue and green), where μ and σ are the mean and standard deviation of the probabilities from training alone. (**Right**) Histogram of the differences between the probabilities of training sequences and the obtained threshold from the testing phase (marked in red line at 0). A false alarm rate of 5.101% is obtained while using this threshold.

With labels, an optimal threshold is obtained from the testing phase using the ROC curve, where the objective function is 100% detection with a minimum false alarm rate. These are the dashed black lines in Figure 3 and Figure 4(left). In order to define a rule of thumb for threshold selection, we describe the relation between the obtained threshold Tr (from the testing phase) and the probabilities of the training sequences alone. Specifically, we referred to the sequences used in the training phase, considering their probabilities from the tree, and examined several levels of thresholds, as shown in Figure 6. We then compared the results with the thresholds (in blue and orange) obtained from the testing phase (blacks in Figure 3 and Figure 4(left)). The thresholds are between the μ+σ and μ+2σ levels (light blue and green lines). Of course, μ and σ were computed without labels. Additionally, in Figure 6, a histogram of the differences between the probabilities of the training sequences and the obtained threshold Tr (marked in red line as zero point) is depicted. According to the results, a false alarm rate of 5.101% is obtained. Namely, the approach discussed above guarantee a false alarm rate close to the one that is shown in Figure 4.

**Figure 7:**
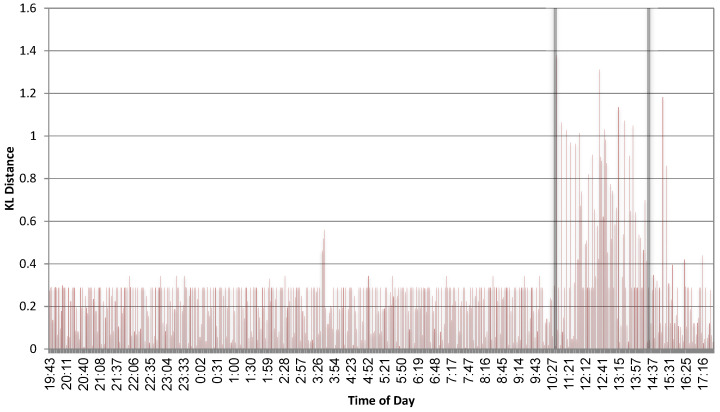
KL distances between the learned histogram of normal behaviour of firefox.exe and the histograms created every two minutes in the testing phase of the same process, as a function of time. The two gray vertical lines mark the time when “Zeus” was active.

Finally, this will also be clear from the results in the next section as well. For example, in Figure 7, when considering all data, including the period where the tool was active, the average is μ=0.46, with σ=0.3. Hence, without any known labeling, setting a threshold slightly above μ+σ will easily identify the period in which the tool was active. Moreover, other approaches for threshold selection were also proposed in [70,71]. We also implemented these in Section 4.1.

## 4. Monitoring the Context of System Calls for Anomalous Behaviour

The sequence of systems calls used by a process can serve as an identifier for the process behaviour and use of resources. When a program is exploited or malicious tools are running, the sequence of system calls might differ significantly as compared to normal behaviour [72]. In this part of the work, the universal anomaly detection tool was used to learn the context of normal system calls, and alert for anomalous behaviour. Specifically, the sequences of system calls created by a process (e.g., firefox.exe) were recorder, processed, and learned. Subsequently, when viewing new data from the same process, the anomaly detection algorithm compared the processed new data to the learned model in order to decide whether the process is still benign, or was it maliciously exploited by some tool.

Due to the large amount of possible system calls, calls were grouped into seven types, based on the nature of the call: Device, Files, Memory, Process, Registry, Security, and Synchronization. That is, unlike the uniform quantization used in Section 3.3, here the calls were grouped based on their functionality. Recording and classification used NtTrace [73]. In the learning phase, system calls were recorder, quantized according to the types above, and then a discrete sequence over the alphabet of size 7 was created. The sequence was used to build the (normal behaviour) LZ tree, as described in Section 2, from which a histogram for the probabilities of tuples of length 20 was calculated. This histogram was the only data saved from the learning phase. The learning phase included four hours of data. For testing, segments of two minutes were recorded. For each segment, a histogram was calculated, similar to the learning phase (calculating probabilities for tuples of length 20 over an alphabet of size 7). Decisions were made based on the KL divergence (also known as the *KL distance*) between the normal histogram and the tested one.

Figure 7 plots the KL distances between the histogram during the learning phase, and the histograms extracted during the testing phase. The process tested was firefox.exe, and the two vertical thick lines mark the time when the tool “Zeus” was active. It is very clear that the context of the system calls changes dramatically when the tool is active, and that simple monitoring of the KL distances every few minutes is sufficient for detecting a change in the system behaviour. However, Zeus, installs instances in several processes simultaneously; hence, it might not be as active within all processes. For example, with winword.exe, it was harder to identify.

### 4.1. An Extreme Value Theory Based Threshold Analysis

For this test case, it is interesting to investigate a different, self-adjusting thresholding mechanism that is based on Extreme Value Theory [70]. This theory deals with the probabilities of rate events, such as threshold exceedances; hence, it is a perfect fit for automatic threshold setting without any labeled data. Specifically, using this method, an initial threshold is set. The initial threshold, *t*, can be chosen based on some estimates from normal data alone. Subsequently, values exceeding this threshold are collected, and fitted to a Generalized Pareto distribution with parameter γ [71]. Denote the best fitting parameter as $\hat{\gamma }$, and the standard deviation of the exceeding points as $\hat{\sigma }$. Denote also by *q* the required exceedance probability. Subsequently, the new threshold is set as

(1) $$t_{new}=t+\frac{\hat{\sigma }}{\hat{\gamma }}\left({\left(\frac{qn}{N_{t}}\right)}^{-\hat{\gamma }}-1\right),$$

where *n* is the total number of samples and $N_{t}$ is the number of exceeding points. For the data in Figure 7, we took t=μ+2σ=0.419 (Based on the statistics for the first two-thirds of the data in Figure 7. Basing it on all the data would result in t=0.64, which would not make a huge difference in the end results). This resulted in $\hat{\sigma }=0.257$. Requiring q=0.01 and setting $\hat{\gamma }=0.1$ concludes with $t_{new}=1.03$. As Figure 7 depicts, this threshold is indeed a better fit than μ+2σ, which is only based on normal data, yet it does not use any labelling.

## 5. Identifying Data Leakage

In this part of the work, the setting was as follows. In the learning phase (a period of a few days), benign traffic on a web server was recorded while using Wireshark [74]. Similar to the previous examples, timing-based sequences were extracted, quantized, and used in order to build an LZ tree. Subsequently, while using Ncat [75], a script was installed on the server. This script initiated downloads of large chunks of data from the server. Several periods, each 30 min. long, of traffic, which includes Ncat, were recorded. For comparison, similar length periods of traffic without Ncat were also recorded. An LZ tree was built for each of the 30 min datasets. To identify data leakage, unlike the Botnets setting considered in Section 3.3; in this case, we compared the joint distributions of *k*-tuples resulting from the LZ trees. That is, we used the distribution of *k*-tuples resulting from the LZ tree as an identifier for the data set, and calculated the distances between the distributions.

Table 2 includes the results. The table depicts the distances between the learned, normal data, and 8 testing periods, 2, which include data leakage using Ncat and 6 without. Two distance measures where used: Mean Square Error (MSE) and KL distance. Under MSE, the leakage sessions clearly stand out as compared to normal data. The results under the KL distance are less clear, especially in the first Ncat session, which included more normal data than the second.

Finally, to further challenge the algorithm, and see whether data leakage will also stand out when the normal communication includes (peaceful) massive downloads, the normal communication was augmented with benign downloads of various sizes. Table 3 depicts the results (under the KL distance). It is clear that, while Ncat stands out when compared to normal traffic on the web server, it is almost indistinguishable when the normal traffic learned includes downloads of large files. This is expected, as Ncat uses a similar protocol, and the key differences in the timing are caused by file sizes. Hence, data leakage is clearly detected compared to normal surfing, yet, it is indistinguishable when the server, in peaceful times, serves large downloads.

## 6. Results and Comparisons on Benchmark Trip Record Data

As a forth test case, we use a completely different data set, which does not involve network or computer security, and is, in fact, entirely man made. Taken from benchmark data sets for anomaly detection [76,77], this widely used data set of one-dimensional time-series includes the number of passengers in NYC taxis from the NYC Taxi and Limousine Commission [78]. Specifically, the basic file used for this work, and many other research projects and anomaly detection commercial packages, consists of a single time series from 2014–2015, with the total number of taxi passengers in 30-min slots [77]. The tested period contains “ground truth” of five known anomalous days [79] (Figure 4d): the NYC marathon (2 November 2014), Thanksgiving (27 November 2014), Christmas (25 December 2014), New Years day (1 January 2015), and a strong New England blizzard (27 January 2015).

The anomaly detection algorithm suggested herein was applied to this data without any significant pre-processing. The raw data were simply uniformly quantized to 20 quantization levels. Subsequently, an LZ tree was constructed based on an initial period (not containing the mentioned anomalies). This completed the learning phase. For testing, the same quantization was done for the new data, and the time series was divided to non-overlapping 10-tuples (5 h of passengers’ data). Each 10-tuple was passed through the tree to compute its probability, and the result was compared to a threshold.

### 6.1. Threshold Computation

Similar to the test case in Section 4, the threshold was set using the EVT approach described in Section 4.1. Specifically, an initial threshold was set based on normal data alone. This time, it was set as μ+2.5σ for the normal data. As this is an initial threshold, and not the one to be finally used, its value does not significantly affect the results. Subsequently, all *above-threshold normal values* were collected, and their histogram was fitted to a Pareto distribution (in this case, since the values correspond to probabilities of 10-tuples, we used the $-\log_{2}\left(\cdot \right)$ values, shifted to start from zero. The fit to the Pareto distribution was done using “EstimatedDistribution” in Mathematica 12). Finally, the threshold to be used in practice is calculated according to (1). Note that, in (1), *q* defines the desired exceedance probability for the new threshold. This value does affect the results, as it affects the number of alarms, either true or false, which one wishes to see.

### 6.2. Results and Comparisons

Setting the desired exceedance probability to 0.5%, a relatively low value results in detecting 4 out of the 5 ground truth anomalous days, with no false alarms at all. A higher value of 2.5%, which corresponds to 5 anomalous days out of a period of about 200 days, results in identifying all ground truth anomalies, with 8 false alarms. With a threshold of $3.13251\times 10^{-15}$ on the probability of 10-tuples, which corresponds to an exceedance rate between the two rates that are mentioned above, the suggested algorithm correctly identified all 5 anomalous days, with only 4 false alarms throughout the entire period.

The results should be compared to [79], with 4 out of 5 anomalies detected and 6 false alarms, or the Numenta [80,81] results with 5 detections and 2 false alarms, and the Prelert [82] results with 5 detections but 9 false alarms [81]. Note that the Numenta algorithm is based on the Hierarchical Temporal Memory network [83]. Roughly speaking, it is a recurrent neural network, yet with a more complex neuron structure and an unsupervised learning rule. Other algorithms reported in [81] have weaker results, as well as the reports in [84] on the same data. Table 4 summarizes these results.

**Figure 8:**
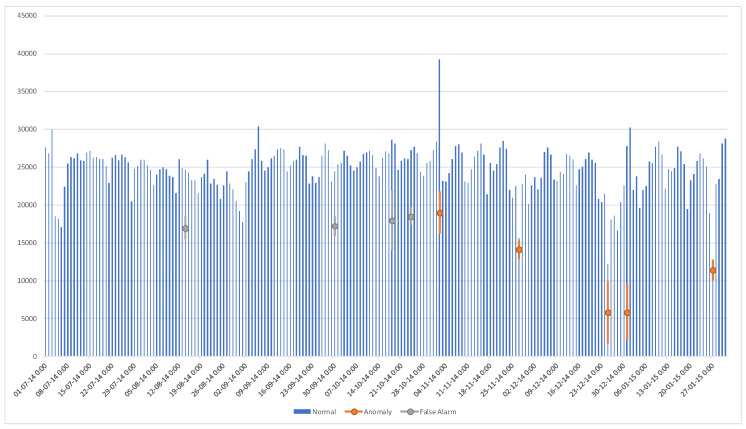
Anomaly detection results for the benchmark file *nyc_taxi.csv*. In blue bars—the number of taxi passengers every 30 min throughout a 6 months period. There data includes 5 known-cause anomalies: the NYC marathon (2 November 2014), Thanksgiving (27 November 2014), Christmas (25 December 2014), New Years day (1 January 2015), and a strong New England blizzard (27 January 2015). All 5 were correctly identified (orange), together with 4 false alarms (gray).

For a graphical representation, Figure 8 includes the raw data, with the identified anomalies in orange, and false alarms in gray. Perplexity computation on the 5 known anomalies results in $1.41938\times 10^{7}$, as compared to $9.50117\times 10^{6}$ for normal data.

## 7. Attack Strategies and Their Consequences

The essence of the technique suggested in this paper is a learning process, which (universally) builds a model for normal data, and then only accepts instances that fit the model. An interesting question is, thus, how easy would it be for an attacker to generate pseudo-normal sequences, such that its traffic will not be identified and blocked? In this section, we offer a few possible strategies, and show that each might result in severe consequences for the attacker, either in terms of huge amounts of data to store, random bits to generate, and long sequences to analyse, or, alternatively, in terms of high probability of error.

### 7.1. The Consequences of an Incorrect Model

First, consider the scenario where an attacker does not have the model that we use for normal data, or access to the true normal data that we learned from. In this case, an attacker wishing to generate data that will be (wrongly) classified as benign needs to use some other statistical model, which he/she assumes is somehow close to the model that we use. Herein, we will show that even a slight difference will cause the attacker to fail.

To simplify the discussion, assume that legitimate sequences are generated i.i.d. according to an underlying probability *P*. While the i.i.d. assumption seems restrictive, the key concept below follows for sequences with memory as well [88]. The attacker does not have access to *P*, hence generates sequences i.i.d. according to some other probability distribution *Q*. *Q* can be close to *P* in some sense, but the two are not equal. The question is then, what is the probability to accept sequences that were generated according to *Q*?

To answer this question, we recall a few definitions and results from the method of types. A type $P_{X}$ of a sequence $X=(x_{1},x_{2},\cdots ,x_{n}$), $x_{i}\in S$, is defined as the relative proportions of occurrences of each element from *S* in *X* (which is a probability mass function on *S*). For example, let S={1,2,3} and X=12123. Accordingly, the type $P_{X}$ is $P_{X}\left(1\right)=\frac{2}{5},P_{X}\left(2\right)=\frac{2}{5}$, and $P_{X}\left(3\right)=\frac{1}{5}$. A type class of $P_{X}$, denoted as $T(P_{X})$, is the set of all sequences of length *n* and type $P_{X}$. Under the above notation, the probability of type class T(Q) under distribution $P^{n}$ is $2^{-nD(Q||P)}$ to the first order in the exponent, or, more precisely [89],

$$\frac{1}{{\left(n+1\right)}^{\left|S\right|}}2^{-nD(Q||P)}\leq P^{n}\left(T\left(Q\right)\right)\leq 2^{-nD(Q||P)},$$

where D(Q||P) is the KL Divergence measure. Consequently, as long as the attacker does not have *P* exactly, and uses an estimate Q≠P, we have D(Q||P)>0, hence $P^{n}\left(T\left(Q\right)\right)$, the probability that our true model *P* will give a sequence from the type class of *Q*, decays exponentially as *n* grows to infinity. As a result, if the anomaly detection system suggested uses long enough sequences in the testing phase, we are guaranteed that the attacker has an exponentially small probability to pass the test, and bypass the detection mechanism.

### 7.2. Using True Data

We now turn to two attack strategies assuming the attacker has some access to true data.

Simple Repeats: assume now the attacker manages to obtain a legitimate sequence, which exists in the LZ78 phrase-tree we use as the model for normal data. Clearly, in the context of Botnets C&C, such a sequence can be available to the attacker by monitoring an HTTP flow of a legitimate connection and extracting the time differences from that session. Obviously, if the attacker tries to use such a sequence periodically, by sending an attack sequence with the same pattern over and over, the attack will fail, as repeating a single sequence, even if it is legitimate and was derived from the true distribution *P*, will create a stream whose distribution is far from *P*. For example, consider a case where one takes a short sequence, say 001 of unbiased coin tosses, and generates 001001001…. Clearly, the resulting sequence will fail a test when compared to an unbiased coin. We will now show that even a more careful technique used by an attacker is still bound by the amount of resources required.

Simulating Sequences from a Single Example: a more sophisticated approach would be to universally simulate a new sequence, or several new sequences, based on the (single) available sequence [90]. The basic idea is as follows. Given a legitimate sequence, considered as a training sequence and denoted by $X^{m}$, where *m* is the length of the sequence, and a string of *k* purely random bits $U^{k}$, which are independent of $X^{m}$, the objective of the attacker is to generate a new sequence of the same length or shorter, denoted as $Y^{n}$, with the same probability distribution as $X^{m}$, yet with minimum statistical dependency between the training sequence and the new one. That is, try to generate new sequences as if we had the generating source itself. To achieve this goal, a deterministic function ϕ(·), independent of the unknown source *P* is employed, such that $Y^{n}=\phi \left(X^{m},U^{k}\right)$, and minimum mutual information $I(X^{m};Y^{n})$ is required in order to guarantee only weak dependence. Fortunately, in order to faithfully represent the characteristics of the data, the input length *m* must be very large, or the number of random bits *k* needed to guarantee low dependency between $X^{m}$ and $Y^{n}$ should grow linearly with the output length *n* [90]. That is, when trying to utilize a single, relatively short training sequence, the attacker will need an unpractical amount of truly random bits.

### 7.3. Accessing the True Model

Finally, one might ask how practical is it for an attacker to build the exact same model used by the anomaly detection system, in order to generate legitimate sequences and disguise illegal activity while using such sequences. First, the attacker must have access to the same database that was used to build the statistical model, and build a tree of practically the same size. However, not only such data might not be available, since, for example, an ISP would not let any third party monitor its entire traffic, the more serious implication is that such a database can be huge, and storing it on a compromised machine (Bot) simply to generate new sequences is not practical. This is because such compromised machines are usually infected using a small email attachment or an infected website, and downloading huge amounts of data will not go undetected for long.

## 8. Conclusions

In this work, we proposed a generic, universal anomaly detection algorithm for one-dimensional time series. The proposed algorithm is based on universal compression and probability assignment, and it is able to build models for the learned data without any prior knowledge or model-assumptions. The models can then be used to detect anomalous behaviour and alert in cases of attacks. The input to the algorithm is a single-feature sequence, usually representing events in time, and feature conversion or normalization is simple scalar quantization.

Specifically, using universal probability assignment techniques that are based on the LZ78 algorithm, we were able to suggest a modeling system that does not require any prior knowledge on the normal behaviour, yet it learns its statistical model optimally, in the sense that it converges to the true probability assignment whenever the source is stationary and ergodic. Together with the optimal decision rule, based on the Neyman—Pearson criteria, the probability assignments result in robust and efficient detection mechanisms. Moreover, as the technique suggested is based on practical universal compression, it can be implemented with low complexity and minimal pre-processing overhead. This should be compared to more sophisticated, recent anomaly detection algorithms, which, while depicting excellent results, use a multi-dimensional feature set, and a relatively complex neural network for learning and testing.

To prove applicability, we applied the algorithms to several problems. The first was detecting C&C channels of Botnets. We evaluated the system on real-world traces, and showed how the context of a simple, single feature, is easily learned using the suggested algorithm, and enables the detection of most Botnets in the data set with negligible false alarm. We continued with additional applications, including the benchmark NYC Taxi data, which concurred with our tests on C&C detection, confirming the applicability of the algorithm. Finally, we showed that any attempt to generate benign sequences to fool the detection system is bound to fail, due to a high probability of error or due to the need for huge amounts of resources.

For future work, a low-complexity, multi-dimensional version of this algorithm can be considered, in order to successfully cope with the multi-feature data that are available in several applications.

## Figures and Tables

**Table 1:** Example for the Database Structure. Each record in the data set represents a data transaction between specific client and specific host/server. Note that in this example, all events started at the same second, yet their duration is different, and the client-server pairs are different.

Time	Time-Taken	Cs-Bytes	Sc-Bytes	Mime-Type	Cat	Hid	Cid
05:52:37	40	803	360	image/gif	good	49	9
05:52:37	74	734	277	text/html	good	102	15
05:52:37	27	578	507	image/gif	good	52	486
05:52:37	27	578	507	image/gif	good	75,526	486
05:52:37	25	655	4196	image/jpeg	good	52	4
05:52:37	25	655	4196	image/jpeg	good	75,526	4
05:52:37	26	577	505	image/gif	good	52	486
05:52:37	26	577	505	image/gif	good	75,526	486
05:52:37	31	624	960	image/gif	good	52	6
05:52:37	1	812	22,672	application/octet-stream	good	75,526	6
05:52:37	30	707	4368	image/jpeg	good	52	2
05:52:37	28	667	2639	image/jpeg	good	75,526	4
05:52:37	180	434	1451	text/html;%20charset=iso-8859-1	hostile	3	6
05:52:37	34	710	4270	image/jpeg	good	52	2
05:52:37	69	697	334	text/css	good	49	9

**Table 2:** Data leakage identification.

	Ncat${}_{1}$	Ncat${}_{2}$	Normal${}_{1}$	Normal${}_{2}$	Normal${}_{3}$	Normal${}_{4}$	Normal${}_{5}$	Normal${}_{6}$
MSE	0.962	1.262	0.044	0.153	0.143	0.43	0.142	0.017
KL	2.05	17.163	1.353	1.228	2.026	4.12	2.121	1.396

**Table 3:** Data leakage identification with additional downloads.

	Normal	Normal + 1.3 MB	Normal + 10 MB	Normal + 200 MB
Normal	0.906	0.843	0.583	0.72
Using Ncat	19.05	0.787	0.733	0.353

**Table 4:** Results on the NYC Taxi data (most assume five anomalies; *—assuming seven anomalies).

Algorithm	True Positive	False Alarm	Miss Detection	Precision	Recall
Numenta [80,83]	5	2	0	0.714	1
The suggested LZ-based algorithm	5	4	0	0.556	1
KNN-CAD [85]	3	4	2	0.429	0.6
Wavelets–based [79]	4	6	1	0.4	0.8
Prelert [82]	5	9	0	0.357	1
TwitterADVec [86], results reported in [87]	4	19	3 *	0.174	0.571

## Abbreviations

The following abbreviations are used in this manuscript:

LZ	Lempel-Ziv
C&C	Command and Control
NYC	New York City
DDoS	Distributed Denial-of-Service
ARMA	Auto Regressive Moving Average
KL	Kullback-Leibler
E-NIDS	Entropy-based Network Intrusion Detection Systems
FSM	Finite State Machine
TD	Time Difference
TT	Time Taken
CSB	Client-Server-Bytes
SCB	Server-Client-Bytes
CID	Client ID (Identification)
HID	Host ID (Identification)
FP	False Positive
TP	True Positive
FN	False Negative
TN	True Negative
ROC	Receiver Operating Characteristic
AUC	Area Under Curve
MSE	Mean Square Error
